# Mitochondrial nucleoid in cardiac homeostasis: bidirectional signaling of mitochondria and nucleus in cardiac diseases

**DOI:** 10.1007/s00395-021-00889-1

**Published:** 2021-08-14

**Authors:** Yuliang Feng, Wei Huang, Christian Paul, Xingguo Liu, Sakthivel Sadayappan, Yigang Wang, Siim Pauklin

**Affiliations:** 1 Botnar Research Centre, Nuffield Department of Orthopaedics, Rheumatology and Musculoskeletal Sciences, Old Road, University of Oxford, Oxford, OX3 7LD UK; 2 Department of Pathology and Laboratory Medicine, Regenerative Medicine Research, University of Cincinnati College of Medicine, 231 Albert Sabin Way, CincinnatiCincinnati, OH 45267-0529 USA; 3 Bioland Laboratory (Guangzhou Regenerative Medicine and Health Guangdong Laboratory), CAS Key Laboratory of Regenerative Biology, Joint School of Life Sciences, Hefei Institute of Stem Cell and Regenerative Medicine, Guangzhou Institutes of Biomedicine and Health, Chinese Academy of Sciences, Guangzhou, 510530 China; 4 Guangzhou Regenerative Medicine and Health Guangdong Laboratory, CAS Key Laboratory of Regenerative Biology, Joint School of Life Sciences, Hefei Institute of Stem Cell and Regenerative Medicine, Guangzhou Institutes of Biomedicine and Health, Guangzhou Medical University, Guangzhou, 510530 China; 5 Guangdong Provincial Key Laboratory of Stem Cell and Regenerative Medicine, Institute for Stem Cell and Regeneration, Guangzhou Institutes of Biomedicine and Health, Chinese Academy of Sciences, Guangzhou, 510530 China; 6 Heart, Lung and Vascular Institute, Division of Cardiovascular Health and Disease, Department of Internal Medicine, University of Cincinnati, Cincinnati, OH 45267 USA

**Keywords:** Mitochondrial nucleoid, 3D genome Organization, Metabolic reprogramming, Cardiovascular diseases

## Abstract

Metabolic function and energy production in eukaryotic cells are regulated by mitochondria, which have been recognized as the intracellular ‘powerhouses’ of eukaryotic cells for their regulation of cellular homeostasis. Mitochondrial function is important not only in normal developmental and physiological processes, but also in a variety of human pathologies, including cardiac diseases. An emerging topic in the field of cardiovascular medicine is the implication of mitochondrial nucleoid for metabolic reprogramming. This review describes the linear/3D architecture of the mitochondrial nucleoid (e.g., highly organized protein-DNA structure of nucleoid) and how it is regulated by a variety of factors, such as noncoding RNA and its associated R-loop, for metabolic reprogramming in cardiac diseases. In addition, we highlight many of the presently unsolved questions regarding cardiac metabolism in terms of bidirectional signaling of mitochondrial nucleoid and 3D chromatin structure in the nucleus. In particular, we explore novel techniques to dissect the 3D structure of mitochondrial nucleoid and propose new insights into the mitochondrial retrograde signaling, and how it regulates the nuclear (3D) chromatin structures in mitochondrial diseases.

## Introduction

**Fig. 1:**
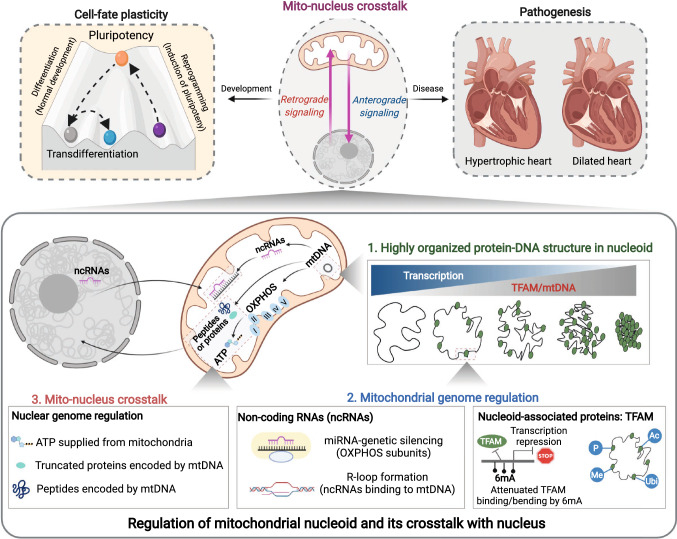
Overview of regulation of mitochondrial nucleoid and its crosstalk with nucleus. Mito-nucleus crosstalk is essential for cell-fate plasticity (differentiation and reprogramming), while its miscommunication is involved in the pathogenesis of heart diseases

Cells can survive in a diversity of environments and reprogram their energy metabolism to adapt to nutritional changes in the microenvironment. This process is commonly referred to as ‘metabolic reprogramming’, and it is an emerging hallmark of the cell-fate decision process [72]. For cellular metabolism and energy production, mitochondria have been recognized as the key hub where metabolic pathways converge and integrate. In particular, this review will focus on mitochondrial metabolism as the primary energy source for the heart. This review will discuss the essential roles of mitochondria in cardiac homeostasis, including cardiac development and differentiation. In addition, mitochondrial dysfunction is a characteristic feature of various cardiac diseases, such as heart failure, owing to an insufficient supply of ATP. It has been well established that transcriptional dysregulation of the mitochondrial genome is central to mitochondrial dysfunction, but the underlying mechanisms are largely unexplored. We further explore the architecture of the mitochondrial genome, its potential regulators, e.g., mitochondrial ncRNA translocated from the nucleus, and how they orchestrate mitochondrial transcription in cardiac metabolic reprogramming. Lastly, we revisit mitochondrial retrograde signaling, its potential impact on nuclear chromatin structures, e.g., 3D chromatin topology, and its implications for mitochondrial diseases, which typically first present with cardiomyopathy (Fig. 1). Collectively, we believe that this review will provide a fresh conceptual look that will allow dissection of the molecular mechanisms of metabolic reprogramming in cardiac diseases.

## Mitochondrial structure and function in cardiac homeostasis

### Mitochondria in heart development

The mitochondrion is a lipid bilayer membrane organelle. It is highly enriched in adult cardiomyocytes which occupy a large portion (approximately 20–30%) of the cytoplasmic space and produce 90% of ATP [193]. Mitochondria contain an outer membrane and inner membrane, two aqueous sub-compartments known as the intermembrane space (IMS) between the two membranes, and the matrix within the inner membrane. The outer membrane is a double phospholipid membrane which is similar to the eukaryotic cell membrane. The outer membrane not only separates the mitochondrion from the cytoplasm, but also contains multiple receptors to mediate communication between mitochondria and other organelles. In addition, some special regions of the outer membrane have been shown to interact with other organelles or other mitochondria, including (i) mitochondria-associated endoplasmic reticulum membranes (MAMs) and (ii) intermitochondrial junctions (IMJs). The intermembrane space contains cytochrome c, which is involved in electron transport and apoptosis regulation. The inner membrane is the site of oxidative phosphorylation (OXPHOS) complexes (complexes I–V) which are involved in electron transport and ATP synthesis. The inner membrane also folds and creates a layered structure named cristae that protrude into the matrix to increases the surface area for energy production. The matrix is enriched in the enzymes and chemicals of the Krebs tricarboxylic acid (TCA) and fatty acid cycle, mitochondrial DNA (mtDNA), ribosomes, enzymes, and ions.

**Fig. 2:**
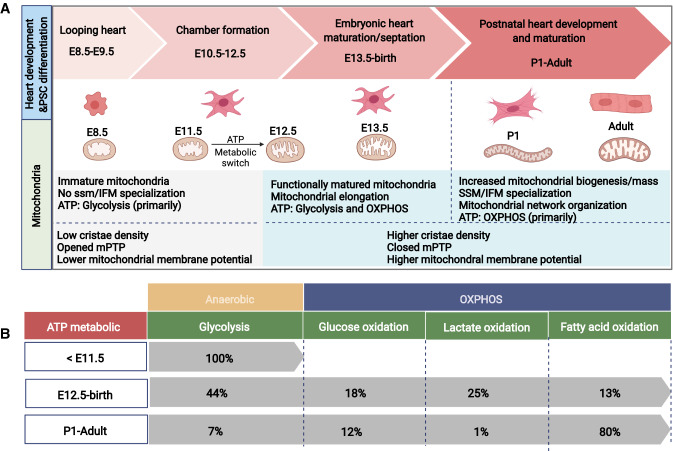
Mitochondrial structure and function in cardiac homeostasis. **A** During heart development and cardiac differentiation, mitochondria are dynamically regulated. This is accompanied by structural differentiation, functional maturation, and metabolic switching; **B** Mitochondrial metabolic features during heart development

During heart development, the mitochondrial system is dynamically regulated to support ATP synthesis, energy transduction, and cell signaling events (Fig. 2). The increase in mitochondrial mass in the growing heart is accompanied by structural differentiation into interfibrillar (IFM) and subsarcolemmal mitochondria (SSM) [43]. These two mitochondrial subpopulations exhibit distinct location, morphology, and functional characteristics. Elongated IFMs are tightly packed into the space between sarcomere Z-lines and lined with sarcoplasmic reticulum, providing ATP for contraction and having higher calcium (Ca^2+^) accumulation. In contrast, the SSMs are located beneath the sarcolemma and provide ATP for active transport of electrolytes and metabolites across the sarcolemma [94, 154]. It has also been reported that the stress response of these two mitochondrial subpopulations are different under pathological conditions, such as cardiac volume [214] and pressure overload [194], hypoxia [87], diabetes mellitus [39, 40], and cardiomyopathy [96]. Metabolic changes also occur during the developing heart. Immature mitochondria can be observed in cardiomyocytes in mice from embryonic day 8.5 (E8.5) to E10.5, and some very immature mitochondria can be observed in cardiomyocytes, as characterized by low cristae density, absence of SSM/IFM specialization, and lactose metabolism. Although OXPHOS complexes can be detected in the inner membrane of mitochondria, cardiomyocytes at these stages primarily acquire ATP through anaerobic glycolysis [180]. Intriguingly, embryonic development will be terminated at E8.5 mouse models lacking essential mitochondrial components [28, 31, 105, 130] and all heart structure [28], suggesting that mitochondria also play a pivotal role in heart development. The number of cristae in cardiomyocytes is increased in mice from E11.5 to E13.5, and many tubular cristae are formed that are connected with the periphery. By E13.5, the functionally matured mitochondria are observed, and cardiomyocytes at this stage acquire ATP through both anaerobic glycolysis and OXPHOS [37, 146]. Noticeably, the mitochondrial permeability transition pore (mPTP) is closed at E13.5, which has been reported to drive mitochondrial elongation, higher mitochondrial membrane potential and cardiomyocyte differentiation in mouse hearts [95]. From E13.5 to prenatal stages, the ATP production pathway shifts from anaerobic glycolysis to aerobic metabolism [69, 146]. At the end of gestation, OXPHOS provides > 50% of ATP in prenatal hearts. Shortly after birth and during the first week of postnatal stages, mitochondrial biogenesis is increased, and the mitochondrial mass of cardiomyocytes is doubled [43, 75]. In addition, the energy metabolism shifts from glucose- and lactate-consuming respiration to the more energy producing β-oxidation of fatty acids [146, 180]. One reason for the change of energy metabolism from glycolysis to OXPHOS in developing heart may be the development of hypoxia inducible factor 1α (HIF-1α) signaling. In the embryonic and fetal (prenatal) heart, HIF-1α is activated in the presence of a hypoxic environment, whereas HIF-1α signaling is decreased in cardiomyocytes after birth [146, 147, 168]. These studies emphasize the importance of mitochondrial homeostasis as essential for embryonic heart development.

### Mitochondria in cardiac differentiation and maturation in pluripotent stem cells

Although mitochondria play an important role in heart development, little is known about their function at the cardiac precursor stage as a result of technical limitations. With the development of efficient cardiac differentiation protocols, the process of cardiac differentiation from pluripotent stem cells (PSCs) is known to recapitulate early cardiac development, thereby providing a potential source for studying otherwise inaccessible cells or tissues. To maintain pluripotency, PSCs mainly depend on glucose and glutamine metabolism. When PSCs exit from pluripotency to the mesoderm, metabolism switches away from glycolysis to OXPHOS which is modulated by several key signaling pathways, including MYC/MYCN transcription inhibition, insulin inhibition, GSK3 inhibition, and Yap inhibition. Upon differentiation, PSCs also display increased numbers and maturation of mitochondria [88]. Studies in PSC cardiac differentiation further suggest that mitochondrial maturation, mitochondrial dynamics, and metabolic shift are prerequisites for the differentiation of stem cells into a functional cardiac phenotype (Fig. 2). In mouse and human PSC-derived cardiomyocytes, the level of mitochondrial OXPHOS and cristae maturation is significantly increased compared to those in PSC, whereas glycolysis is decreased [37]. Inhibition of OXHOS during cardiac differentiation prevents mitochondrial organization, causing poor cardiomyocyte differentiation, as characterized by deficient sarcomerogenesis and contractile malfunction [37]. Consistent with mouse developing heart, the fragmented mitochondrial network in PSCs progressively fuses and elongates during differentiation, which further confirms the essential role of mitochondrial fusion in proper cardiomyocyte differentiation [112]. mPTP inhibition by Cyclosporin A (CsA) facilitates the differentiation of functional cardiomyocytes from mouse and human PSCs [35]. Moreover, the differentiated cardiomyocytes display unique metabolic features in contrast to non-cardiomyocytes. PSC-derived non-cardiomyocytes proliferate and mainly rely on glucose and glutamine metabolism; however, differentiated cardiomyocytes rely on lactate oxidation [211, 212]. Understanding the metabolic cues in heart development not only facilitates cardiac differentiation, but also enables the development of nongenetic methods to purify PSC-derived cardiomyocytes for clinical applications [88].

### Mitochondrial regulation of cardiac diseases

**Fig. 3:**
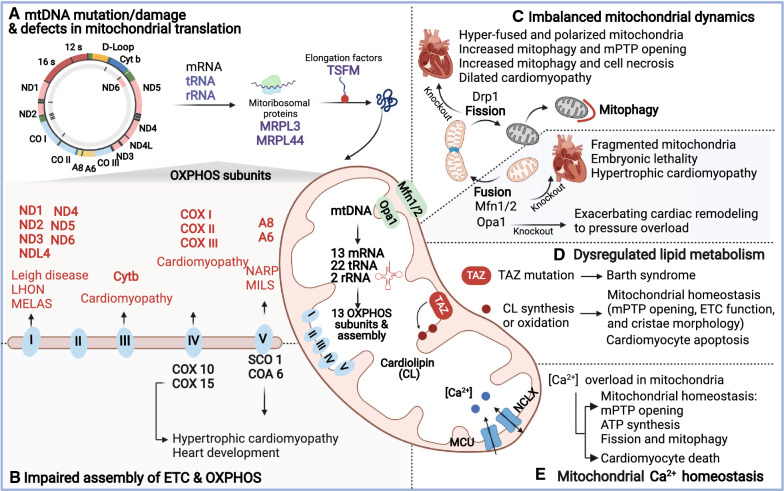
Mitochondrial regulation of cardiac diseases. Schematic view of major mitochondrial dysregulation implicated in progressive decline of myocardial structure and function, including mitochondrial DNA (mtDNA) mutation/damage (red color) and defects in mitochondrial translation (purple color) (**A**), impaired assembly of ETC and OXPHOS (blue color) (**B**), imbalanced mitochondrial dynamics (**C**), dysregulated lipid metabolism (**D**), and mitochondrial Ca^2+^ overload (**E**). *LS* Leigh syndrome, *LHON* Leber hereditary optic neuropathy, *MELAS* mitochondrial encephalomyopathy, lactid acidosis and stroke-like episodes, *NARP* neurogenic muscle weakness, ataxia and retinitis pigmentosa, *MILS* maternally inherited Leigh syndrome

Accumulating evidence has demonstrated the central role of mitochondria in the preservation of cardiac homeostasis. Major theories of mitochondrial dysregulation, including mitochondrial DNA (mtDNA) mutation/damage, impaired OXPHOS, defects in mitochondrial translation, imbalanced mitochondrial dynamics, dysregulated lipid metabolism, and mitochondrial Ca^2+^ overload, are all associated with progressive decline in myocardial structure and function (Fig. 3). The mtDNA has a very high rate of mutation [162, 185], but only limited repair capacity [245]. Therefore, mtDNA mutation and inadequate mtDNA level compromise the integrity of the mitochondrial genome, resulting in defects in electron transport chain (ETC) complexes and ATP supply. Various mtDNA mutations have been detected in heart tissues. Genetic defects in ETC structure subunits of the OXPHOS system (complex I, II or III) [5, 7, 29, 161] and proteins associated with proper assembly of respiratory chain complexes [30], such as mutation in COX10 and COX15 or the assembly factors of complex IV [8], have been described in different types of cardiomyopathy. Recently, pathogenic mutations in components of the mitochondrial translation machinery have also been linked to various progressive human diseases, including neurological disorders, heart/muscle diseases, and endocrine problems [127]. During translation of mitochondrial mRNAs, various translation factors interact with the mitochondrial ribosome to synthesize the polypeptides. Mitochondrial RNA contains two mitochondrial ribosomal RNAs (12S mt-rRNA and 16S mt-rRNA) and 22 mt-tRNAs encoded by mtDNA. Mutation of mt-rRNAs [52, 145, 192], mt-tRNAs [4, 74, 76], mitochondrial ribosomal proteins (MRPL3 and MRPL44), and the translation elongation factor (TSFM) [1, 48, 54, 65] have all been documented in various cardiomyopathies, together with decreased synthesis of mitochondrial polypeptides and impaired energy production. As highly dynamic organelles, homeostasis of mitochondria is tightly controlled by a quality control system consisting of fission, fusion, and degradation of mitochondria, collectively referred to as mitochondrial dynamics. Fusion involves content exchange, allowing complementation of mitochondrial solutes, proteins and DNA. Fission allows segregation of damaged mitochondria eliminated by selective degradation (mitophagy). Mitochondrial dynamics is essential for mitochondrial shape, size, distribution, quality control (mitophagy), and transport of mitochondria within the cell. For example, when cells are challenged by stress, mitochondrial dynamics spring into action to trigger compensatory and adaptive cellular response [49, 176]. Pathogenic mutations in the core machinery and defects of mitochondrial dynamics-related proteins have been documented in various cardiac diseases. Recently, mitochondrial lipid metabolism was found to play an important role in the regulation of mitochondrial dynamics [70]. Although most lipids are synthesized in the endoplasmic reticulum (ER), the mitochondrial membrane also contributes to phospholipid synthesis, including cardiolipin (CL), phosphatidic acid, and phosphatidylethanolamine (PE) [157]. Mitochondrial lipid metabolism has been associated with cardiomyopathy in Barth syndrome (BTHS), an x-linked autosomal recessive disease [103]. The underlying cause of BTHS is the mutation of CL transacylase tafazzin (TAZ), which results in the impaired synthesis of CL and compromises mitochondrial homeostasis. CL is a highly abundant lipid in heart tissues and is important in maintaining mitochondrial homeostasis, ranging from mitochondrial protein transport, mitochondrial dynamics, mitochondrial cristae formation, and respiratory chain to cytochrome c-related apoptosis [51]. CL directly interacts with optic atrophy 1 (Opa1), a core component of mitochondrial fusion machinery [70], and it is also associated with the processing of Opa1 [172]. Impaired Opa1 processing has been reported to cause dilated cardiomyopathy and heart failure [221], indicating an intriguing involvement of CL-regulated Opa1 processing. Interestingly, mtDNA-protein complexes were found to be co-fractionated with mitochondrial lipid components [71]. Further research has shown that mtDNA stability and segregation are associated with mitochondrial lipid metabolism [148]. Recent studies of mitochondrial Ca^2+^ overload have revealed a critical role in the pathophysiology of heart failure [240]. Specifically, excessive Ca^2+^ in mitochondria will increase the generation of ROS and mPTP opening, thereby causing mitochondrial dysfunction and cell death [163]. No direct evidence has suggested that mtDNA polymorphisms/mutations are involved in dysregulated mitochondrial Ca^2^ in heart diseases. Nevertheless, it has been documented that mtDNA polymorphisms alter mitochondrial calcium levels in forebrain cells of mutant mtDNA polymerase (Polg) transgenic mice [114]. These studies put the mitochondrial genome squarely in the center of the cardiac homeostatic network which is responsible for coordinating the involvement of mitochondria in cellular homeostasis.

## Higher organization of the mitochondrial genome

### The mitochondrial genome forms highly organized protein–DNA structures in the nucleoid

Mitochondria are endosymbiotic organelles containing their own genome, mtDNA. The mtDNA, as an ‘extranuclear’ genetic molecule, is unique and different from nuclear DNA (nDNA), the regulation of which is independent from nDNA. The mtDNA is generally packaged into nucleoids, the heritable units of mtDNA. The organization of mtDNA is compact without intronic structures in protein-coding genes and often contains overlapping reading frames between neighboring genes, thereby leading to higher gene density in mtDNA than that in nDNA [243]. Although the size of mtDNA is small, the mRNA transcribed from mtDNA can represent a surprisingly large proportion of total cellular mRNA, nearly 30% in the heart [159]. Mammalian mtDNA is a compact circular DNA molecule of 16,569 base pairs with a contour length of 5 µm that possesses coding regions (> 90%, around 93% in humans and mice) and noncoding regions. The coding regions encode 11 mRNAs (translated to critical proteins of the OXPHOS), 2 ribosomal RNAs (rRNAs, 12S and 16S), and 22 transfer RNAs (tRNAs). The noncoding regions include D-loop and the light-strand origin of replication (OriL) that harbors regulatory regions for mtDNA transcription and replication.

Unlike nDNA, mtDNA lacks histones and is packaged into the nucleoid, as noted above, inside the mitochondria matrix, the only known organization unit of the mitochondrial genome containing mtDNA and associated architectural proteins, thus forming a distinct protein-DNA structure. Mammalian mitochondrial nucleoids are slightly ellipsoid with an average shape varying between slightly elongated (80 × 80 × 100 nm) [125] to truly ellipsoid (25 × 45 × 100 nm) [3, 24]. Because of its extranuclear localization, mtDNA was thought to follow a maternal pattern of inheritance with no paternal contribution [99, 222]. However, the discovery of Will Luo and colleagues presents an interesting conceptual breakthrough in that mtDNA can also be occasionally paternally inherited [149]. The number of mtDNA molecules per nucleoid is currently debated among several researchers. Recent studies support the idea of multiple mtDNA copies, ranging from 1.4 to 7.5 mtDNA per nucleoid [133], challenging the idea of a single copy of mtDNA per nucleoid [125]. However, the nucleoid morphology and size are independent of mtDNA copy number [125]. In mouse embryonic fibroblast cells (MEFs), an increase in mtDNA copy number induced by human TFAM overexpression leads to an increase in the total number of nucleoids per cell and cell size when compared with the wild-type MEFs, while the nucleoid size is fundamentally unaffected [125]. As revealed by proteomic analyses, a large number of potential mitochondria nucleoid-associated proteins have been identified and can be grouped into different functional classes. Apart from the key components of mitochondrial transcription (POLRMT, TFAM, TFB2M, TEFM) and replication (POLG, Twinkle, mtSSB) machinery, other important proteins, such as mitochondrial ribosomal proteins, proteases, chaperones, RNA-binding proteins, and RNA-processing proteins, have also been associated with the nucleoid. It was recently suggested that the mitochondrial nucleoid, not the mtDNA, is a unit of mitochondrial genome inheritance [68]. Nucleoids are semiregularly spaced within mitochondria to secure correct mtDNA transmission into the daughter cells at cell division [108, 173]. In addition, nucleoid-associated proteins, such as TFAM, have also been reported to influence mtDNA transmission [2, 113]. Knockdown of TFAM not only results in an abnormal nucleoid, but also causes asymmetric transmission of mtDNA into two daughter cells [113]. Thus, significant interest in nucleoid structure and its regulation has arisen to address the underlying mechanisms of mitochondrial diseases.

### Mitochondrial nucleoid-associated proteins

As the most abundant architectural protein in the mitochondrial nucleoid, TFAM plays a major role in nucleoid structure. TFAM, a DNA-binding protein with tandem high-mobility group (HMG)-box domains, is highly conserved across species and present at a ratio of 1000 molecules per mtDNA, or 1 subunit per 16–17 bp of mtDNA [21]. When bound specifically to mtDNA promoters or nonspecifically throughout the mtDNA genome, TFAM fully coats and packages mtDNA into nucleoids by creating a stable U-turn with an overall bend of 180° [169]. TFAM is essential for the maintenance of mtDNA integrity, including mtDNA packaging [116, 170], transcription [196], replication [56], mtDNA copy number maintenance [101], and correct transmission [2, 113]. Growing evidence obtained from TFAM knockout mice has established a critical role of TFAM in the regulation of mitochondrial function and cardiac homeostasis. Inactivation of TFAM by Nkx2.5Cre in the mouse embryonic heart at E7.5 induces disrupted mitochondrial biogenesis and morphology, elevates ROS production, and depolarizes mitochondria, thereby causing embryonic lethality at E15.5 with marked myocardial wall thinning [248]. Other cardiomyocyte-specific TFAM knockout mouse models further characterized the effect of TFAM inactivation on mitochondrial cardiomyopathy with the development of dilated cardiomyopathy (DCM) [225]; [134, 202]. A decrease of TFAM expression has also been observed in several cardiac failure models with mitochondrial dysfunction [67, 100, 115, 131]. On the contrary, overexpression of TFAM exhibited therapeutic potential in heart failure, leading to the reduction of protease expression, ROS production, cytoplasmic calcium [128], and attenuated pathological hypertrophy [64] in cardiomyocytes. Cardioprotective effects were further confirmed in transgenic mouse models containing human TFAM gene, as evidenced by ameliorated mitochondrial deficiencies and improved cardiac function after myocardial infarction [102]. Although TFAM inhibition efficiently decreased the levels of mitochondria-encoded transcripts [104, 225], notably, overexpression of TFAM did not affect mtRNA levels, but rather mtDNA copy number [102]. These lines of evidence imply the presence of more complex regulatory mechanisms responsible for the association of TFAM, mtDNA, and mitochondrial homeostasis.

In fact, different mtDNA compaction from fully compacted nucleoids to naked DNA has been observed from the effect of physiological variation in TFAM on mtDNA ratios [60]. At high TFAM:mtDNA ratios, the nucleoid is fully compacted such that TFAM forms large, stable filaments on the DNA in a manner that blocks DNA melting through POLRMT and TWINKLE, thus preventing active mtDNA replication and transcription [60]. In support of this notion, a mild increase in TFAM levels in vivo (∼twofold) leads to a proportional increase in mtDNA copy number [53], whereas forced overexpression of TFAM leads to mtDNA depletion [178]. Interestingly, when mtDNA was depleted in the presence of ethidium bromide (EB), knockdown of Lon (the regulator of TFAM) is unable to degrade TFAM, thereby causing a dramatic increase in the TFAM:mtDNA ratio, in turn resulting in the inhibition of mitochondrial transcription [156]. This indicates that the TFAM:mtDNA ratio may affect the number of compacted mtDNA and open mtDNA molecules.

Considering the role of TFAM as a histone-like protein in mtDNA packaging, it is plausible that TFAM also has modifications equivalent to those that occur on nuclear histones. In the nucleus, histones pack nDNA into nucleosomes forming chromatin. Modification of histones, including phosphorylation, methylation, acetylation, and ubiquitylation, strongly influences gene integrity [117]. Recently, nuclear-encoded transcription factors that regulate nuclear chromatin were found in the mitochondrial nucleoid, including MOF [32], members of the AP1 family (c-Jun and JunD), CEBPB [20] and MEF2D [195]. Most importantly, MOF, a histone lysine acetyltransferase that remodels chromatin, was found to directly bind to mtDNA and inhibit mtDNA transcription [32]. Interestingly, TFAM levels were not obviously affected in these MOF-depleted cells. The discovery of TFAM-DNA-binding affinity, as regulated by acetylation and phosphorylation of TFAM [120], raises the question of whether TFAM is the acetyltransferase target of MOF in mitochondria. Moreover, TFAM was found to facilitate damaged mtDNA degradation by accelerating strand cleavage at abasic (apurinic/apyrimidinic, AP) sites [187], a ubiquitous form of DNA damage [141]. Considering the full coating of mtDNA by TFAM, these results suggest the ubiquitous nature of the TFAM-mediated abasic site containing mtDNA degradation.

For a long time, the higher-order organization of mtDNA in the nucleoid was considered far less regulated and complex owing to the lack of chromatin and histones. Intriguingly, recent analysis of DNase-seq and ATAC-seq experiments from multiple human and mouse samples revealed the formation of a conserved footprint during embryonic development, as an indication that mtDNA sites are increasingly occupied, but have low TFAM occupancy [19, 153]. Moreover, mtDNA sites with low occupancy of TFAM tend to adopt noncanonical nucleic secondary structures known as G-quadruplex sequences (GQs) [19]. GQs are notably conserved across species, and GQ motifs have been increasingly recognized to be associated with epigenetic reprogramming and chromatin remodeling [216]. GQs are widely present in mtDNA, as revealed by recent studies [55, 98], and GQ formation potentially regulates mitochondrial gene transcription and replication [55, 91, 167]. These studies further reflect the existence of the higher-order organization of protein-DNA structure in the mitochondrial genome in which the accessibility of mtDNA is potentially regulated by the TFAM:mtDNA ratio. Development of efficient experimental tools that allow selective detection of mitochondrial GQs or isolation of mitochondrial GQ structures could facilitate the discovery of novel mtDNA-binding proteins. Although important progress has been made in deciphering the molecular mechanisms of mito-nuclear communication in protein-DNA interaction, mitochondrial genome regulation is more complex and more regulated than once thought. Hence, it will be important for future studies to decipher the structure and organization of nucleoids and their specialized functions.

### Mitochondrial genome regulation by noncoding RNAs

The discovery of noncoding RNAs (ncRNAs) has extended our knowledge of the molecular pathways involved in cardiac homeostasis. NcRNAs, as a large part of the noncoding genome, play various roles in cellular processes, rather than serving as a protein template. NcRNAs are classified into three types based on their localization and genetic origin: (1) cytoplasmic, nuclear-encoded ncRNAs; (2) mitochondria, nuclear-encoded ncRNAs and imported to the mitochondria; (3) mitochondria, mtDNA-encoded ncRNAs. Recently, ncRNAs have been increasingly found in the mitochondria and termed as mito-ncRNAs. These ‘mito-ncRNAs’ are ncRNAs located inside mitochondria, irrespective of their genetic origin [217]. Significant research has been aimed at unveiling how the ncRNAs are linked with their specific subcellular localizations and functions. These mito-ncRNAs can be grouped into microRNAs [miRNAs, 17–23 nucleotides (nt)], long noncoding RNAs (lncRNAs, > 200 nt), and circular RNAs (circRNAs). Cytoplasmic ncRNAs can modulate mitochondrial functions through a cytoplasmic mechanism. Next, we will review the role and function of particular mito-ncRNAs in cardiac homeostasis (see Table 1).

**Table 1:** Mitochondrial ncRNAs in cardiac diseases

Name	Targets	Function	Phenotype	References
miR-1	COX1 and ND1 MCU	Enhanced expression of mitochondrial COX1 and ND1 Mitochondrial Ca^2+^ homeostasis	Inhibition of miR-1 ameliorates cardiac hypertrophy/heart failure Cardiac hypertrophy	[90] [109, 247]
miR-20	Mfn2	Mitochondrial fusion	Cardiac hypertrophy	[207]
miR-30c	Complex I (NDUFB8), complex II (SDHB), complex III (UQCRC2), complex IV (MTOC1), and HSP60	Mitochondrial OXPHOS	Dilated cardiomyopathy	[232]
miR-106a	Mfn2	Mitochondrial fusion	Cardiac hypertrophy	[82]
miR-142-3p	SH2B1	Mitochondrial membrane potential and mitochondrial density	Upregulation of miR-142-3p ameliorates cardiac hypertrophy	[142]
miR-485-5p	Mfn2	Mitochondrial fusion	Cardiac hypertrophy	[253]
miR-497	Sirt4	Mitochondrial membrane potential and cytochrome c release	Overexpression of miR-497 inhibits cardiac hypertrophy	[238]
miR-34a	ALDH2	Mitochondrial alcohol metabolism	Activation of miR-34a promotes cardiomyocyte apoptosis post-myocardial infarction	[57]
miR-28	ALDH2	Mitochondrial alcohol metabolism	Activation of miR-28 promotes cardiomyocyte apoptosis and ischemic injury	[136]
miR-762	Complex I (ND2)	Mitochondrial ATP, ROS, and complex I enzymatic activity	Upregulation of miR-762 promotes cardiomyocyte apoptosis and ischemic injury	[241]
miR-181c	Complex IV (COX1)	Complex IV remodeling and ROS	Upregulation of miR-181c promotes ROS level in neonatal cardiomyocytes	[42]
miR-195	Sirt3	Regulation of mitochondrial function (mitochondrial acetylome)	Heart failure	[250]
miR-665	GLP1R, cAMP signaling pathway	Complex I-IV	Inhibition of miR-665 ameliorates heart failure	[139]
miR-499	Pdcd4, Pacs2, and Dyrk2	Mitochondrial-related apoptosis (inhibition of Bid expression and mitochondrial translocation)	Upregulation of miR-499 protects H_2_O_2_-induced injury in cardiomyocytes	[224]
miR-3453p miR-532 miR-690 miR-696	Unknown	Unknown	These miRNAs are upregulated in the mitochondria of failing hearts	[227]
LncRNA LIPCAR	Unknown	Unknown	Circulating levels of mitochondrial lncRNA LIPCAR are downregulated early after acute myocardial infarction and upregulated during later stages and are associated with adverse cardiac remodeling and death, which predicts survival in heart failure patients	[126]
CircRNA MFACR	Targets miR-652-3p and the gene encoding mitochondrial membrane protein MTP18	Mitochondrial fission and apoptosis	Downregulation of circRNA MFACR attenuates fission and heart failure post-myocardial infarction	[226]

#### Mitochondrial miRNAs

As the most studied ncRNAs, miRNAs are short endogenous ncRNA species that are highly conserved. They recognize the complementary sequences of their message RNA (mRNA) target and form the RNA-induced silencing complex (RISC), thereby controlling gene expression post-transcriptionally by regulating mRNA degradation or translation [150]. However, miRNAs located in mitochondria (mito-miRs) are unique and distinguished from cytosolic miRNAs which are characterized by (1) unusual size (17–25 nt *vs*. average 22 nt length for the cytosolic miRNAs); (2) preferential genomic localization in mitochondrial gene clusters or close to mitochondrial genes (genomic position) [10, 11]; (3) unique structural features, such as containing no or small 3′UTR, short 3′overhangs, stem-loop secondary structures [218], only one part of RNA-induced silencing complex (RISC), and multiple binding sites on mtDNA, but lacking 5′ cap [84]; and (4) unique thermodynamic features, e.g., minimal folding free energy (MEF) [10, 11]. These unique features of mito-miRs have been speculated to facilitate not only their entry into mitochondria but also their interaction with mtDNA transcripts.

The first evidence of miRNAs identified in mitochondria was described by Kren et al. in 2009 [123]. They found 15 nuclear-encoded mito-miRs isolated from adult rat livers as evidenced by Northern blot and stem-loop RT-PCR analyses. Similarly, Barrey et al. (2011) further confirmed the presence of miRNAs, including pre-miR-let7b, pre-miR-302a, and their corresponding mature miRNAs in mitochondria, as assessed by RT-PCR and in situ hybridization [12]. The presence of these pre-miRNAs in mitochondria might presuppose that the miRNA process occurs in mitochondria. This would also mean that the mitochondrial genome can encode miRNAs, but this hypothesis needs further investigation. Given the well-characterized functional role of mitochondria in ATP production, it is not surprising that mito-miRNA-mediated OXPHOS activity is associated with heart diseases. Among these miRNAs involved in heart failure development, miR-1 is not only the most abundant miRNA in the heart, but it is also a muscle mitochondrial-enriched miRNA. The expression of miR-1 is downregulated in infarcted hearts [22], and it regulates mitochondrial ETC by directly regulating multiple proteins in the ETC networks, including cytochrome c oxidase subunit 1 (mt-COX1) and the mitochondrial gene NADH dehydrogenase subunit 1 (mt-ND1) [251]. Notably, in this model, miR-1 acts as a mitochondrial translation activator whereby AGO2 can bridge the ribosome (12SrRNA) to the miR-1-bound mtRNA to activate translation [251]. MiR-1 is also involved in mitochondrial Ca^2+^ overload through targeting the mitochondrial calcium uniporter (MCU) mRNA and inhibiting its translation, thereby leading to cardiac hypertrophy [109, 247]. Furthermore, miR-181c, as the mitochondrial-located miRNA in cardiomyocytes [42], was shown to cause mitochondrial and cardiac dysfunction [41, 42]. Through their ability to modulate mitochondrial function, metabolism and dynamics, mito-miRs have been implicated in cardiac homeostasis, as summarized in Table 1.

#### Mitochondrial lncRNAs

Although most studies have focused on miRNAs in cardiac homeostasis, research has also explored the function and biological significance of lncRNAs in cardiac homeostasis. LncRNAs, as a new class of regulatory ncRNAs, are longer than 200 nt and evolutionarily less conserved compared to miRNAs. The first evidence of mito-lncRNA was described by Beckham et al. in 2011 [182]. They discovered that three lncRNAs (lncND5, lncND6, and lncCytb) specifically produced by the mitochondrial genome and their accumulation in mitochondria is regulated by MRPP1. Mitochondrial-encoded lncRNAs are divided into three categories: (1) simple antisense mitochondrial DNA-encoded lncRNAs (e.g., lncND5, lncND6, lncCytb, lncRNA, and MDL1) [66, 159, 182], (2) Chimeric mtDNA-encoded lncRNAs (e.g., SncmtRNA, ASncmtRNA-1, and ASncmtRNA-2) [25, 129, 220], and (3) putative mtDNA-encoded lncRNAs (e.g., LIPCAR) [47, 126, 242]. Interestingly, only limited evidence supports the presence of nuclear-encoded lncRNAs in mitochondria owing to the unresolved mitochondrial import mechanism. Among the lncRNAs implicated in cardiac dysfunction, only one mito-lncRNA has been reported. The circulating mitochondrial lncRNA uc022bqs.1 (LIPCAR) was found to be upregulated in the late stages of patients who developed left ventricular remodeling post-myocardial infarction. With another independent cohort of 344 patients with chronic heart failure, the level of LIPCAR was upregulated independent of pathogenesis. Most importantly, higher LIPCAR levels were significantly associated with a higher risk of cardiovascular death [126]. The importance of mito-lncRNAs in the pathogenesis of cardiomyopathy was further supported by recent studies that reveal a distinct relative abundance of dysregulated lncRNA of mitochondrial origin in failing hearts. These lncRNAs encoded by mtDNA constitute the majority (71%) of the total lncRNA pool of the human left ventricle [242]. Hence, it not clear if mito-lncRNAs detected in circulation come from heart. Moreover, these lncRNA profiles can better distinguish samples of cardiomyopathy with different etiology/mechanical circulatory support than either mRNA or miRNA profiles. Thus, mito-lncRNAs could be useful for the detection and diagnosis of disease subtypes in the future.

#### Mitochondrial circular ncRNAs

As the least investigated ncRNAs, circular ncRNAs (circRNAs) present a closed circle structure resulting from a covalent bond between their 3′ and 5′ ends. Although the function of more than 99% of circRNAs remains unclear, recent studies have revealed their roles in miRNA and RBP sponging, transcriptional and post-transcriptional regulation, protein templates, and immune response [158, 219, 234]. The first evidence of circRNAs in mammalian mitochondria was described by Gao et al. in 2018. Three mitochondria-encoded circRNAs were found, but their functional information was not further investigated [66]. In 2019, Liu et al. further identified hundreds of circRNAs encoded by the mitochondrial genome in both human and murine cells. Strikingly, they first found that two circRNAs encoded by the mitochondrial genes ND1 and ND5 could facilitate the mitochondrial entry of nuclear-encoded proteins through the TOM40 complex [144]. This discovery broadened our understanding of the critical roles of nuclear-encoded protein mitochondrial shuttling. Although mtDNA-encoded circRNAs have been identified [110, 144, 191], the functions of mitochondria-localized circRNAs (mito-circRNAs) are largely unknown as a result of the special mitochondrial bilayer structure and highly negative membrane potential [228]. Recently, mito-circRNAs have attracted extensive attention in human diseases. A circulating mito-cirRNA, mc-COX2, was found to be highly expressed in chronic lymphocytic leukemia (CLL) patients and significantly associated with its progression and prognosis [236]. Most recently, a mitochondrial-enriched circRNA, ATP5B regulator (SCAR), was screened by circRNA expression profile analysis of patients with nonalcoholic steatohepatitis (NASH). In patients with NASH, SCAR was downregulated in liver fibroblasts, an outcome closely correlated with disease progression. Overexpression of SCAR in NASH fibroblasts blocked mPTP opening, inhibiting mitochondrial ROS output and subsequent fibroblast activation. In mice fed a high-fat diet, specific delivery of SCAR to liver fibroblasts using mitochondria-targeting nanoparticles alleviates cirrhosis and insulin resistance accompanied with NASH [252].

Although no direct evidence currently supports the association between mito-cirRNAs and cardiac homeostasis, one circRNA (MFACR) has been reported to directly target miR-652-3p and promote mitochondrial fission and cardiomyocyte apoptosis through blocking the translation of MTP8 [226].

#### Potential impact of ncRNA on mitochondrial R-loop formation

The ncRNAs are increasingly found in mitochondria, but their functions remain largely unknown. Recently, R-loop in mitochondria (termed as mtR-loop) was surprisingly detected [244] and associated with mitochondrial genome stability [199]. R-loops are three-stranded nucleic acid structures comprising a RNA:DNA hybrid and an unpaired single-stranded DNA. In general, R-loops occur transiently during transcription, playing essential physiological functions. However, when R-loops occur accidentally in a non-scheduled manner, they become a potential source of cellular pathological R-loop. A growing body of evidence has connected these pathological R-loops to the pathophysiology of diseases [81], including DNA repair, replication, activation of tumor-promoting genes [18, 21, 121, 201], and immune response [152]. Recent studies showed that lncRNAs can interact with DNA and form R-loop structures [33, 80]. In addition, Fan et al. reported that nuclear-derived microRNA (miR-2392) can be shuttled into mitochondria and hybridize with mtDNA, leading to downregulation of the genes responsible for oxidation phosphorylation, metabolic reprogramming, and chemoresistance in cancer [58]. These pioneering studies suggest that mitochondrial ncRNA may regulate mitochondrial transcription through the formation of R-loops.

An important question is whether ncRNAs can interact with DNA to form R-loop structures. To address this, it is necessary to establish an assay suitable for R-loop mapping in mitochondria. DRIP-seq is the first and widely used approach for genome-wide mapping of R-loop in vitro by harnessing S9.6 antibody for chromatin immunoprecipitation [73]. However, the resolution for DRIP-seq is low (around several kilobases) from the fragmentation of genomic DNA by restriction enzyme and thus cannot satisfy the investigation of R-loop in mitochondria with only 16.5 kb mtDNA. In addition, the specificity of S9.6 has been recently questioned since, for example, it also binds dsRNA/dsDNA [177], leading to false positivity. RNaseH1 is an endonuclease which displays high affinity of RNA–DNA hybrids and cleaves the RNA in RNA:DNA hybrids.

**Fig. 4:**
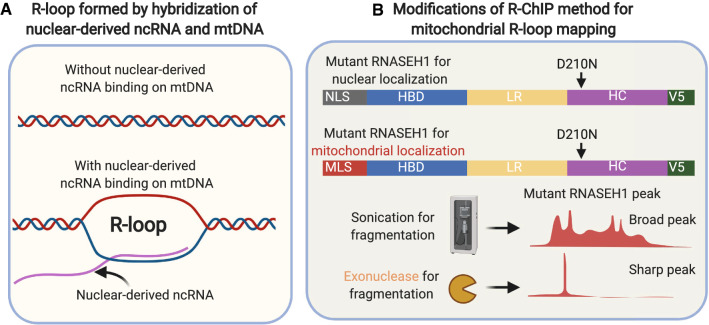
Novel directions for mito-nucleus crosstalk studies: the potential impact of ncRNA on mitochondrial R-loop formation. **A** Mitochondrial localized ncRNAs hybridize mtDNA to form R-loop structures. **B** Modifications of R-ChIP method to dissect R-loop structures in mitochondria: **1** Replacement of nuclear localization signal (NLS) with mitochondrial localization signal (MLS) to direct mutant RNaseH1 (D210N) into mitochondria. **2** Harnessing exonuclease, rather than sonication, for fragmentation of mutant RNaseH1/R-loop complex to improve resolution

Recently, the Xiang-dong Fu lab developed an R-ChIP [33, 34] approach using catalytically inactive RNaseH1 (point mutation in the catalytic site (D210N)) with the addition of a nuclear localization signal (NLS) in the N-terminus and a tagged V5 sequence at the C-terminus. Followed by sonication, instead of restriction enzyme, as in DRIP-seq, chromatin immunoprecipitation (anti-V5 antibody), library preparation and sequencing, this approach can detect genome-wide R-loops in the nucleus with high resolution of around 200–300 bp. We propose that this method is ideal for R-loop studies in mitochondria when minor modifications are made, such as replacement of NLS with the mitochondrial localization signal (MLS), to direct the RNaseH1 (D210N) into mitochondria. Furthermore, the resolution for mitochondrial R-loop could be further increased using exonucleases, rather than sonication, during the fragmentation step, which is similar to the ChIP-nexus protocol [89], to obtain the “footprint” of RNaseH1 (D210N). Such high-resolution mapping can help locate the actual R-loop structures in the “busy” mitochondrial genome harboring 37 genes and many regulatory elements within 16.5 kb DNA (Fig. 4).

Emerging evidence suggests that mitochondrial damage-associated molecular patterns (DAMPs) are the major activators of inflammation when leaked from stressed mitochondria, which then facilitates cardiac dysfunction [77, 111, 174, 205]. Of the known mitochondrial DAMPs, mitochondrial nucleic acids, such as mtDNA, have garnered the most attention [23, 230]. Cardiomyocyte-derived mtDNA, having escaped from defective mitophagy, could trigger inflammation, thereby inducing myocarditis and dilated cardiomyopathy [171]. Although numerous independent studies have supported the inflammatory nature of ‘cell-free mtDNA’ [45, 46, 213, 246], this set of experiments does not distinguish between the presence of double-strand DNA (dsDNA, mtDNA) versus R-loop. Thus, the strategies used to characterize the mtR-loop are important and will ultimately enable novel therapeutic regimens for disease intervention where R-loop regulation is dysfunctional.

Recently, a class of small proteins, or peptides (micropeptides), encoded by short open reading frames (sORFs) within ncRNAs (e.g., lncRNAs), are now being recognized for their fundamental biological importance in ion channel modulation [6], cell signaling [155] and RNA regulation [38]. Most importantly, these micropeptides are surprisingly enriched in mammalian mitochondrial proteome, accounting for 5% of its proteins [27]. One example is mitoregulin (MOXI, MPM), a muscle- and heart-enriched 56 amino acids encoded by mito-lncRNA (linc00116) [36, 140, 151, 204]. Mitoregulin localizes within inner mitochondrial membrane, binds cardiolipin, and influences protein complex assembly and/or stability, thereby regulating mitochondrial respiratory (super) complex formation and activity, fatty acid oxidation, TCA cycle, and Ca^2+^ dynamics, putatively explaining how mitochondrial homeostasis is regulated by ncRNAs.

## Three-dimensional (3D) organization of mitochondrial nucleoid

Recent mapping technologies have revealed that the eukaryotic genome in the nucleus does not exist as a linear molecule, but instead is packaged into a 3D structure with higher-order organization. The 3D chromatin organization in the nucleus enables the regulation of gene expression during cardiac development in both physiological processes in health and pathogenesis of disease [16, 188]. As discussed above, mtDNA is compacted into a nucleoprotein complex, forming a higher-order chromatin-like organization for transcription regulation. Hence, it would be interesting to unravel the 3D architecture of mitochondrial nucleoid and examine the existence of higher-order architecture in the mitochondria similar to topologically associating domains (TADs) in the nucleus.

**Fig. 5:**
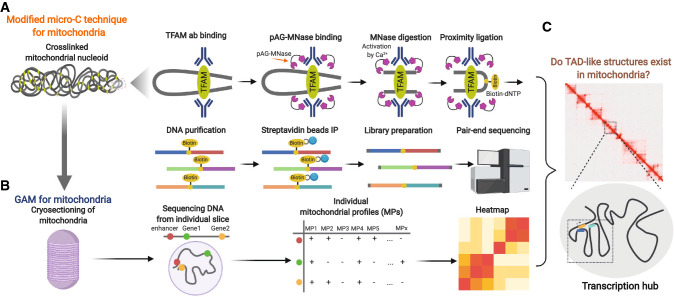
Novel strategies to dissect 3D genome structure of the mitochondrial nucleoid. **A** Modified micro-C approach to map TFAM-centric 3D genome structure of mitochondria (pAG-MNase: protein A/G beads conjugated with MNase); **B** Genome architecture mapping (GAM) approach to map global 3D genome structure of mitochondria via cryosectioning, sequencing of DNA from individual slices and computational analysis (co-segregation frequency); **C** 3D genome mapping of mitochondrial nucleoid can examine the existence of topologically associating domains (TADs) and transcriptional hub (interaction of multiple regulatory elements) in the mitochondrial nucleoid

In the past few years, 3D genome approaches (e.g., Hi-C) in the nucleus have used restricted enzymes (e.g., HindIII [138], MboI [183], and DpnII [14]) for chromatin fragmentation, followed by chromatin ligation. The resolution of Hi-C depends on the frequency of the restricted enzyme recognition sites and the sequencing depth. In most publications, the maximal resolution of Hi-C is around several kilobases on average in the genome. Considering the distribution of restricted enzyme recognition sites on the mitochondrial genome with small size (about 16.5 kb), the conventional Hi-C approach is not suitable for the dissection of 3D architecture of the mitochondrial nucleoid. This calls for the development of novel techniques that enable us to examine the interaction of regulatory elements in mitochondria and also examine the existence of TAD-like structures in the nucleoid. Recently, two breakthrough publications demonstrated that a new technique called micro-C can detect micro-scale genome organization up to single-nucleosome resolution in human [97, 124]. Unlike Hi-C using restricted enzymes, micro-C uses MNase which first acts as an endonuclease by nicking the DNA on the A/T site and then functions as an exonuclease to nibble the DNA until it meets the protein “block” (e.g., histone or transcription factor) [206]. In the micro-C method, the MNase-digested nucleosomes are kept for downstream proximity-ligation reaction. However, mitochondria lack nucleosomes, making this protocol unusable in mitochondrial samples. Here, we propose modifications of micro-C that would make it applicable to the mitochondrial genome. Currently, protein A/G-conjugated MNase is employed in the CUT and RUN method [200] to capture histone or transcription factor binding sites. In the CUT and RUN method, a primary antibody is added to the nucleus after cell permeabilization to target the chromatin. Then protein A/G-MNase is added into the nucleus. It recognizes the sites with primary antibody binding and can, therefore, cut chromatin enriched with the specific protein, i.e., the target of primary antibody. Since TFAM is thought to be the major constituent for mitochondrial nucleoid organization with the capacity of DNA bending and compaction, the TFAM antibody and protein A/G-MNase could be used to isolate the TFAM-DNA complex, followed by ligation and other steps in the micro-C protocol. This modified micro-C approach would allow for dissection of the TFAM-centric 3D architecture of the mitochondrial nucleoid (Fig. 5A). In addition to TFAM, other nuclear-derived chromatin modifiers/transcription factors (e.g., MOF [32], AP-1 [20], CEBPB [20], and MEF2D [195]) are reported to be involved in the regulation of mitochondrial transcription, suggesting their potential contributions in mitochondrial nucleoid organization. Thus, for an unbiased discovery of the 3D architecture of mitochondrial nucleoid, a recent approach called genome architecture mapping (GAM) could be utilized [13]. GAM was developed to measure the 3D genome organization via sequencing the DNA from a large quantity of nuclear sections and statistical modelling. Similarly, GAM would be performed in mitochondria. Through cutting thinner slices, it could be possible to obtain a high-resolution map of 3D architecture of mitochondrial nucleoid (Fig. 5B). In addition, compared to proximity ligation-based approaches, such as Hi-C, which can only detect binary interactions, i.e., one locus to one locus, GAM is a ligation-free approach and thus can identify the transcription hub, or interactome of multiple regulatory elements, in the mitochondrial genome. These approaches would help us to demystify the existence of TAD (topologically associating domains)-like structures in mitochondria and further unveil the transcription regulation of metabolic reprogramming in health and disease (Fig. 5C).

## Emerging regulators for 3D architecture of mitochondrial nucleoid

N6-Methyldeoxyadenosine (6 mA) is the major DNA modification in prokaryotes [215]. However, whether 6 mA exists in mammalian genomes remains a top controversial issue in epigenetic research. This mainly results from the low abundance and large variation of 6 mA distribution in different mammals and different tissues (0.0001% ~ 1%) [93, 122, 143, 237, 239], raising several questions about its biological functions. Through ultra-high performance liquid chromatography coupled to triple quadrupole mass spectrometry (UHPLC-QQQ-MS/MS), immunofluorescence and dot blot experiment, the Chuan He lab found that 6 mA is highly enriched in human mtDNA and that the abundance is 1300-fold higher than that of genomic DNA in a human liver cancer cell line [85]. The deposition of 6 mA on mtDNA is orchestrated by mitochondria-localized METTL4, a putative mammalian methyltransferase, and the authors showed that 6 mA impaired DNA binding and bending mediated by TFAM, leading to transcriptional repression of mitochondria. Furthermore, under hypoxia, METTL4 and m6A are significantly elevated compared to normoxia. These data suggest that the METTL4/6 mA could sense and respond to metabolic stress via modulation of the 3D organization of mitochondrial nucleoid [85]. A previous report demonstrated that the abundance of 6 mA was highly dynamic during the embryonic development of *Drosophila* (∼0.07% at the ∼0.75 h. *vs*. ∼0.001% at the 4 h) [249]. Thus, whether 6 mA on mtDNA is highly dynamic during human embryonic development, resulting in dynamic alterations of 3D architecture of mitochondrial nucleoid and metabolic reprogramming, deserves future investigation.

Interestingly, a recent study suggested that mitochondrial nucleoids might be formed by phase separation. The authors found that the combination of TFAM-mtDNA is sufficient to form gel-like droplets in vitro, which are affected when intrinsically disordered regions (IDR) of TFAM are mutated [61]. This is in line with previous studies from the Richard Young lab, showing that IDR is essential for transcription factor coactivators BRD4 and MED1 to form droplets (compartmentalization) and, thus, facilitate gene transcription [189]. The authors also showed that enlarged phase-separated mitochondrial nucleoids could be observed in the premature ageing disease Hutchinson-Gilford Progeria Syndrome (HGPS) in association with TFAM and that this is linked to impaired mitochondrial oxidative phosphorylation and ATP regeneration [61]. However, the underlying mechanisms for enlarged phase-separated mitochondrial nucleoids in HGPS remain elusive. Besides the potential alteration of IDR in TFAM, the impact of 6 mA on phase separation would be another potential mechanism to be validated. Recent study has uncovered that N6-Methyladenosine (m6A) on RNA can enhance phase separation [186]. Therefore, the potential of 6 mA affecting 3D architecture of mitochondrial nucleoid via modulation of phase separation would be an intriguing research direction in the future.

Mitochondrial retrograde signaling and its potential impact on nuclear 3D chromatin architecture

Hierarchical 3D genome organization includes multiscale structural units of chromosome territories, compartments, topologically associating domains (TADs), which are often demarcated by such chromatin architectural proteins as CCCTC-binding factor (CTCF) and cohesin, and chromatin loops [254]. However, the mechanisms underlying the formation and function of nuclear chromatin architecture have not been fully resolved. Notably, mitochondria can generate a wide range of retrograde signals through which they regulate nuclear gene expression. Here we discuss the implication of mitochondrial retrograde signals for 3D genome organization and propose possible strategies to test this.

### Mitochondrial-derived ncRNAs

Although mitochondrial-generated ncRNAs (e.g., ASncmtRNA-1 and ASncmtRNA-2) are translocated into the nucleus and associated with perinuclear chromatin [62, 129], their functions in transcription regulation remain largely unknown. Recently, four approaches, termed global RNA Interactions with DNA by deep sequencing (GRID-seq) [137], Chromatin-Associated RNA sequencing (ChAR-seq) [15], HiChIRP [164], and RNA in situ conformation sequencing (RIC-seq) [26], have identified that chromatin-associated RNAs (CARs) are largely involved in long-range chromatin interactions. For example, ncRNA can bind to the *MYC* enhancer and *MYC* promoter and interact with the RNA-binding protein hnRNPK, while the oligomerization of hnRNPK can juxtapose the enhancer to *MYC* promoter and promote *MYC* transcription [26]. In addition, ncRNA *ThymoD* facilitates CTCF/cohesin-dependent loop extrusion and repositions *Bcl11b* enhancer from nuclear periphery proximal to *Bcl11b* promoter, leading to transcription activation of Bcl11b and T cell commitment [106].

**Fig. 6:**
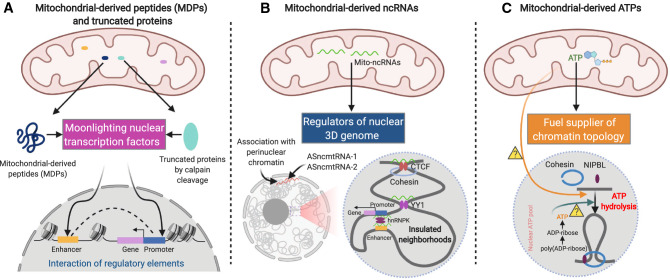
Novel directions for mito-nucleus crosstalk studies in cardiac diseases: mitochondrial retrograde signaling and its potential impact on nuclear 3D chromatin architecture. **A** Mitochondrial-derived peptides or truncated proteins (cleaved by mitochondrial calpain) that moonlight as transcription factors to act on nuclear regulatory elements and induce their interactions for transcription regulation; **B** Mitochondrial-derived ncRNAs regulate nuclear 3D genome organization via association of perinuclear chromatin, orchestration of insulated neighborhoods (binding on architecture proteins CTCF and YY1), and facilitation of enhancer-promoter interaction (one binding on enhancer/promoter; two interacting with RNA-binding protein hnRNPK; three oligomerization of hnRNPK to juxtapose enhancer and promoter); **C** Loop extrusion is mediated by Cohesin and NIPBL through ATP hydrolysis, while the source of ATP, whether mitochondrial, nuclear pool by hydrolysis of poly(ADP-ribose) to ADP-ribose, or both, remains to be determined in the future

In addition to juxtaposing enhancers and promoters in the 3D genome, CARs can also modulate chromatin topology. For example, YY1 is an emerging architectural protein for orchestrating enhancer-promoter interaction [229], and its localization on DNA is facilitated by direct binding with CARs [198]. Furthermore, insulated neighborhoods are chromatin topological structures (loops) formed by CTCF-CTCF homodimers [50] and the major structural components of TADs, the boundaries of which are enriched by CTCF [92]. CTCF can bind large numbers of ncRNA, and a recent report from Danny Reinberg’s lab indicated that mutation of the RNA-binding region of CTCF could disrupt CTCF loop formation, resulting in transcriptional inactivation [190]. This means that the functions of ncmtRNAs in CTCF formation could be further delineated in the future. In addition, whether nuclear-localized ncmtRNAs regulate genome compartment (e.g., heterochromatin) formation and maintenance through m6A-mediated phase separation [186] will be another intriguing topic for further investigation (Fig. 6A).

### Mitochondrial-derived peptides or truncated proteins

Although the vast majority of mitochondrial proteins are encoded in the nucleus, some new classes of peptides were recently discovered with mitochondrial origins. These mitochondrial-derived peptides (MDPs) are the peptides encoded by small open reading frames (ORFs) within mtDNA (mtORF) [223]. At present, three types of MDPs have been identified, including 16S rRNA-encoded Humanin (HN), 12S rRNA-encoded MOTS-c and 16S rRNA-encoded small humanin-like peptides (SHLP1-6). As the first MDP, HN was discovered by Hashimoto and his colleagues in the undamaged brain of a patient with Alzheimer’s disease [86]. HN structure and function has been well studied, and various HN analogs are believed to have protective effects against cardiovascular diseases [78] in addition to neuronal diseases [132, 208]. Administration of an analog of HN (the serine at position 14 replaced by glycine, termed as HNG) prior to, or at the time of, reperfusion has been shown to play a cardioprotective role in a mouse model of myocardial ischemia and reperfusion, which potentially improved cardiomyocyte survival and attenuated apoptosis [166]. Similarly, high-doses of HNG applied during the ischemic period also exerted cardioprotection, leading to significantly decreased cardiac arrhythmia, myocardial infarct size, apoptosis, cardiac mitochondrial dysfunction, and left ventricular dysfunction [209]. Under pathologic conditions, increased ROS is detrimental to cardiomyocytes [179]. Specifically, complexes I and III of the ETC are major sites of ROS production in cardiac mitochondria [203]. Recent studies confirmed the contribution of HNG to reduced ROS production in damaged cardiac mitochondria. Administration of HNG both activated the Abl- and Arg-dependent cellular defense system and decreased complex I activity [210]. Additionally, cardioprotective effects of HNG were recently found in aged hearts through preventing myocardial fibrosis, mPTP opening, and apoptosis [181]. Further studies also show the relationship between MDPs and coronary microvascular dysfunction (CMD), which are both risk factors for cardiac dysfunction. In the cohort of patients with CMD, lower levels of HN were observed in circulating blood [231]. Another independent study also found that the levels of HN and MOTS-c in circulating blood of patients with impaired endothelial function were lower than those of the control groups [181]. These results suggest that HN or MOTS-c has significant potential as a new marker to diagnose CMD or identify potential therapeutic targets.

Recent research has reported that MOTS-c translocates from mitochondria into the nucleus in response to glucose restriction, leading to retrograde signaling [118, 184]. MOTS-c can bind to nuclear genomic DNA and interact with transcription factor Nrf2 to activate gene transcription, resulting in increased cellular resistance to metabolic stress [118]. Reynolds et al. in 2021 found that MOTS-c is an exercise-induced mitochondrial-encoded regulator of muscle homeostasis which can regulate the nuclear genes involved in metabolism and proteostasis, thereby significantly improving physical performance in young, middle-aged, and old mice [184]. Moreover, administration of MTOS-c at late-life (23.5 months) can increase the lifespan of old mice. These landmark studies provided a new paradigm for understanding the impact of MDPs on nuclear gene regulation in metabolic reprogramming.

In addition, a recent study surprisingly found that calpain can cleave full-length junctophilin-2 to covert it from a cardiac structural protein into a transcription factor, leading to its translocation from the cell membrane into the nucleus for cardiac protection [83]. As calpain is not only localized in cytosol, but also in mitochondria [9, 107, 175, 197], future work is needed to delineate the proteins that shuttle from mitochondria into nucleus after mitochondrial-calpain cleavage and subsequently regulate chromatin architecture (Fig. 6B).

### Mitochondrial-derived ATP could be essential fuel for the establishment of topological domains in the nucleus

The loop extrusion model is the most popular model for CTCF loop, insulated neighborhood formation and TADs [63, 92]. This model postulates that cohesin complex is loaded onto DNA by physical stimulation and reels in DNA to form loops. Loop extrusion can then be stopped when cohesin meets convergent CTCF sites. Recent studies have provided direct evidence in support of this model and have shown conclusively that cohesin extrudes DNA loop in an ATP hydrolysis-dependent manner [44, 119]. Previously, it was well recognized that ATP is generated only from mitochondria. However, this prevailing “dogma” was recently challenged, showing that ATP can also be generated in the nucleus via the hydrolysis of poly (ADP-ribose) to ADP-ribose and that this was shown to be essential for hormone-induced chromatin remodeling in a breast cancer cell line [235]. This raises several questions. First, is the “nuclear pool” only confined to breast cancer cells or present in all mammalian cells? Second, what is the source of ATP for loop extrusion, mitochondria, nucleus or both? Most mitochondrial diseases are caused by mutations of mtDNA, leading to defects in the OXPHOS system and reduction of ATP production [79] and cardiac involvement [233]. Therefore, third, does ATP reduction caused by mutations of mtDNA cause global loss of CTCF loops and transcriptional dysregulation?

Precise mtDNA editing was long deemed as impossible. However, a recent study developed a novel approach based on RNA-free DddA-derived cytosine base editors (DdCBEs), which can catalyse nucleotide conversions in human mtDNA with high target specificity [160]. Based on this technology, human iPSC-derived cardiomyocytes from patients with diseases like hypertrophic cardiomyopathy [135], before and after mtDNA editing (rectification of mtDNA mutation), can be generated and used to study disease mechanisms. For example, by performing high-resolution 3D genome mapping (e.g., in situ Hi-C or CTCF/Cohesin-centric in situ ChIA-PET [17]; HiChIP [165]; or PLAC-seq [59]) in the nucleus from those cells, it is possible to assess the impact of mtDNA mutation on 3D genome reprogramming in the nucleus of cardiomyocytes (Fig. 6C). Such studies would give completely fresh insight into the pathogenesis of mitochondrial diseases with cardiac involvement and reveal the plausible impact of mtDNA mutation on transgenerational inheritance of abnormal nuclear genome topology in patients.

## Closing remarks

A growing body of research has established that mitochondria serve as the core component of the cell signaling pathway. Unveiling the mechanisms that control how mitochondrial nucleoid components orchestrate cell responses to stressful stimuli is essential for a better understanding of the transition between cardiac health and disease. Researchers have only just begun to understand how communication between mitochondria and nucleus are coordinated in response to metabolic stress. In this review, we have taken a fresh perspective on 3D genome architecture and its role in the crosstalk between mitochondria and nucleus. We believe that studies designed to demystify the overlooked questions delineated in this review will provide a foundational understanding of the regulation of metabolic reprogramming and its translation into clinical benefits in the future.

## Data Availability

Not applicable.

## Abbreviations

CARs: Chromatin-associated RNAs
CMD: Coronary microvascular dysfunction
CTCF: CCCTC-binding factor
CsA: Cyclosporin A
circRNAs: Circular ncRNAs
DAMPs: Damage-associated molecular patterns
DdCBEs: DddA-derived cytosine base editors
DCM: Dilated cardiomyopathy
ETC: Electron transport chain
GQs: G-quadruplex sequences
HIF-1α: Hypoxia inducible factor 1α
HGPS: Hutchinson–Gilford progeria syndrome
IMJs: Intermitochondrial junctions
IMS: Intermembrane space, m6A, N6-methyladenosine
MAMs: Reticulum membranes
MDPs: Mitochondrial-derived peptides
mPTP: Mitochondrial permeability transition pore
mtDNA: Mitochondrial DNA
ncRNAs: Noncoding RNAs
6MA: N6-methyldeoxyadenosine
Opa1: Optic atrophy 1
OXPHOS: Oxidative phosphorylation
PSCs: Pluripotent stem cells
RISC: RNA-induced silencing complex
SSM: Subsarcolemmal mitochondria
TADs: Topologically associating domains
TSFM: Translation elongation factor
